# Crystal structure of 4,4-dibutyl-2-phenyl-3,4-di­hydro­quinazoline

**DOI:** 10.1107/S1600536814020017

**Published:** 2014-09-10

**Authors:** Gamal A. El-Hiti, Keith Smith, Amany S. Hegazy, Mohammed B. Alshammari, Benson M. Kariuki

**Affiliations:** a Cornea Research Chair, Department of Optometry, College of Applied Medical Sciences, King Saud University, PO Box 10219, Riyadh 11433, Saudi Arabia; b School of Chemistry, Cardiff University, Main Building, Park Place, Cardiff CF10 3AT, Wales; c Chemistry Department, College of Sciences and Humanities, Salman bin Abdulaziz University, PO Box 83, Al-Kharij 11942, Saudi Arabia

**Keywords:** crystal structure, quinazoline, hydrogen bonding

## Abstract

In the title compound, C_22_H_28_N_2_, the dihedral angle between the planes of the phenyl ring and the di­hydro­quinazoline ring system (r.m.s. deviation = 0.030 Å) is 24.95 (7)° and both *n*-butane chains assume all-*trans* conformations. In the crystal, N—H⋯N hydrogen bonds link the mol­ecules into *C*(4) chains propagating in the [001] direction.

## Related literature   

For the synthesis of 4,4-dibutyl-2-phenyl-3,4-di­hydro­quin­azo­line, see: Smith *et al.* (2005 ▶); Plé *et al.* (1997 ▶). For the crystal structures of related compounds, see Valkonen *et al.* (2011 ▶); Derabli *et al.* (2013 ▶).
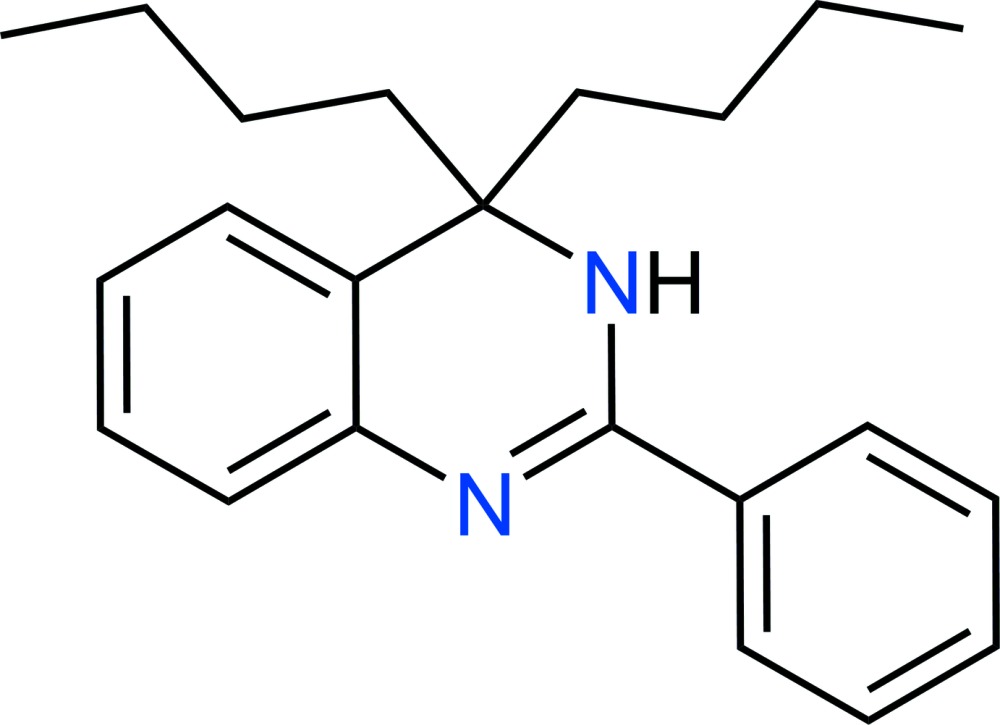


## Experimental   

### Crystal data  

C_22_H_28_N_2_
*M* *_r_* = 320.46
Monoclinic, 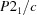

*a* = 19.2953 (8) Å
*b* = 9.9889 (3) Å
*c* = 9.6341 (4) Å
β = 96.667 (4)°
*V* = 1844.31 (12) Å^3^
*Z* = 4
Cu *K*α radiation
μ = 0.51 mm^−1^
*T* = 150 K
0.41 × 0.13 × 0.04 mm

### Data collection  

SuperNova, Dual, Cu at zero, Atlas diffractometer
Absorption correction: multi-scan (*CrysAlis PRO*; Agilent, 2014 ▶) *T*_min_ = 0.829, *T*_max_ = 1.000
12894 measured reflections
3657 independent reflections
2866 reflections with *I* > 2σ(*I*)
*R*_int_ = 0.043

### Refinement  

*R*[*F* ^2^ > 2σ(*F* ^2^)] = 0.047
*wR*(*F* ^2^) = 0.129
*S* = 1.04
3657 reflections
219 parameters
H-atom parameters constrained
Δρ_max_ = 0.23 e Å^−3^
Δρ_min_ = −0.17 e Å^−3^

### 

Data collection: *CrysAlis PRO* (Agilent, 2014 ▶); cell refinement: *CrysAlis PRO*; data reduction: *CrysAlis PRO*; program(s) used to solve structure: *SHELXS97* (Sheldrick, 2008 ▶); program(s) used to refine structure: *SHELXL2013* (Sheldrick, 2008 ▶); molecular graphics: *ORTEP-3 for Windows* (Farrugia, 2012 ▶) and *CHEMDRAW Ultra* (Cambridge Soft, 2001 ▶); software used to prepare material for publication: *SHELXL2013*.

## Supplementary Material

Crystal structure: contains datablock(s) I, New_Global_Publ_Block. DOI: 10.1107/S1600536814020017/hb7281sup1.cif

Structure factors: contains datablock(s) I. DOI: 10.1107/S1600536814020017/hb7281Isup2.hkl

Supporting information file. DOI: 10.1107/S1600536814020017/hb7281Isup3.cml

**Fig. 1.**
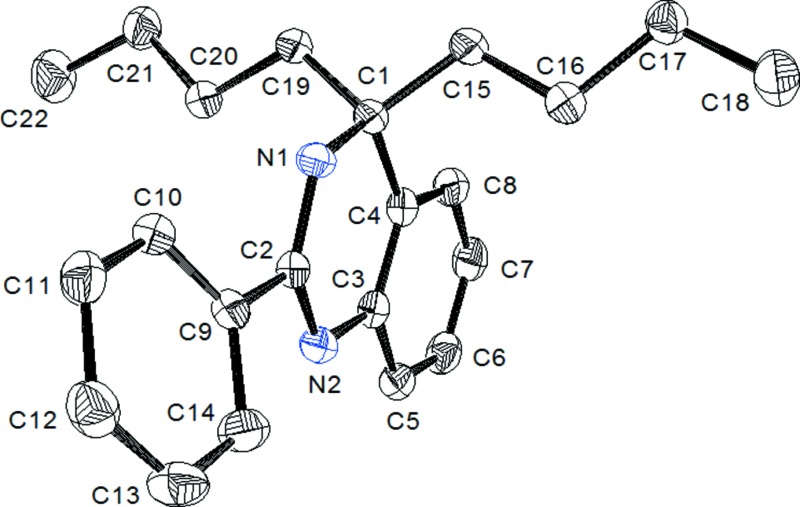
The asymmetric unit of the title compound with 50% probability displacement ellipsoids.

. DOI: 10.1107/S1600536814020017/hb7281fig1.tif
The asymmetric ubit of the title compound with 50% probability displacement ellipsoids.

**Fig. 2.**
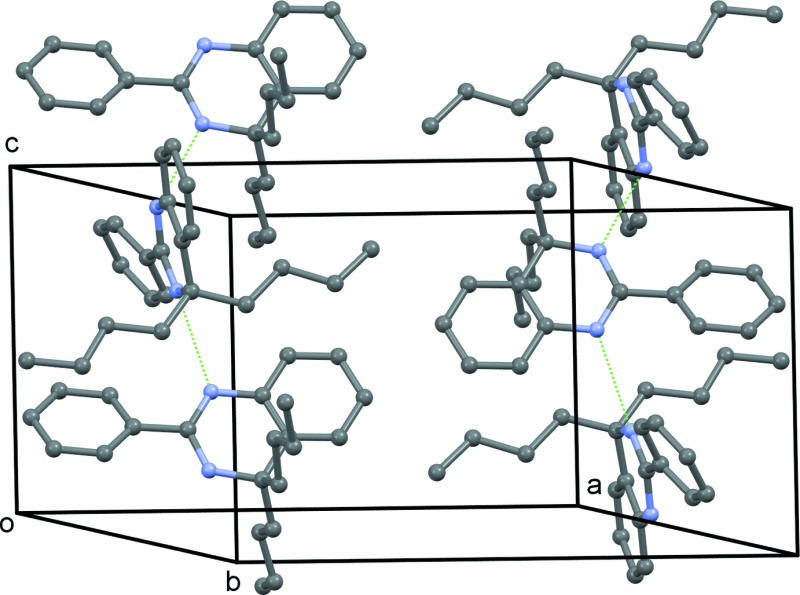
Packing in the crystal structure showing N—H···N contacts as dotted lines with hydrogen atoms omitted for clarity.

. DOI: 10.1107/S1600536814020017/hb7281fig2.tif
Packing in the crystal structure showing N—H⋯N contacts as dotted lines with hydrogen atoms omitted for clarity.

CCDC reference: 1022964

Additional supporting information:  crystallographic information; 3D view; checkCIF report

## Figures and Tables

**Table 1:** Hydrogen-bond geometry (Å, °)

*D*—H⋯*A*	*D*—H	H⋯*A*	*D*⋯*A*	*D*—H⋯*A*
N1—H1⋯N2^i^	0.88	2.29	3.1239 (16)	157

Symmetry code: (i) 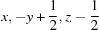.

## supplementary crystallographic information

### S2. Structural commentary

In the molecule of C_22_H_28_N_2_ (Fig. 1), the phenyl ring is twisted by 24.95 (7) from the plane of the di­hydro­quinazoline group. Both *n*-butane chains assume all-*trans* conformation. N—H···N hydrogen bonds between neigbouring molecules form chains parallel to the *c*-axis (Fig. 2).

4,4-Di­butyl-2-phenyl-3,4-di­hydro­quinazoline can be obtained from reaction of two mole equivalents of *n*-butyl­lithium with 4-(methyl­thio)-2-phenyl­quinazoline at –78°C in anhydrous THF [Smith *et al.* (2005); Plé *et al.* (1997)]. The reaction involves initial addition of *n*-butyl­lithium at the 4-position of quinazoline ring followed by elimination of methane­thiol­ate anion and then further addition of *n*-butyl­lithium (Smith *et al.*, 2005). For the X-ray structures of related compounds, see Valkonen *et al.* (2011); Derabli *et al.* (2013).

### S5. Synthesis and crystallization

4,4-Di­butyl-2-phenyl-3,4-di­hydro­quinazoline

A solution of *n*-butyl­lithium in hexane (1.76 mL, 2.5 M, 4.4 mmol) was added to a cold (–78 ^ο^C), stirred solution of 4-(methyl­thio)-2-phenyl­quinazoline (0.50 g, 2.0 mmol) in anhydrous THF (10 mL) under N_2_. The reaction mixture was stirred at –78 ^ο^C for 1 h then removed from the cooling bath and allowed to warm to room temperature, diluted with di­ethyl ether (10 mL), then quenched with aqueous saturated NH_4_Cl (10 mL). The organic layer was separated, washed with water (2 x 10 mL), dried (MgSO_4_), and evaporated under reduced pressure. The residue obtained was purified by column chromatography (silica gel; di­ethyl ether–hexane, 1:4 by volume) to give 4,4-di­butyl-2-phenyl-3,4-di­hydro­quinazoline in 96% yield, m.p. 161 ^ο^C [lit. 161 ^ο^C: Smith *et al.* (2005); 154–155 ^ο^C: Plé *et al.* (1997)]. Crystallization from a mixture of ethyl acetate and di­ethyl ether (1:3 by volume) gave the title compound as colorless crystals. The spectroscopic data for the title compound, including NMR and low and high resolution mass spectra, were consistent with those reported [Smith *et al.* (2005)].

### S6. Refinement

H atoms were positioned geometrically and refined using a riding model, with *U*_iso_(H) constrained to be 1.2 times *U*_eq_ for the bonded atom except for methyl groups where *U*_iso_(H) was 1.5 times and free rotation about the C—C bond was allowed. Crystal data, data collection and structure refinement details are summarized in the table.

### Crystal data

**Table:**

C_22_H_28_N_2_	*F*(000) = 696
*M**_r_* = 320.46	*D*_x_ = 1.154 Mg m^−^^3^
Monoclinic, *P*2_1_/*c*	Cu *K*α radiation, λ = 1.5418 Å
*a* = 19.2953 (8) Å	Cell parameters from 3898 reflections
*b* = 9.9889 (3) Å	θ = 4.6–73.6°
*c* = 9.6341 (4) Å	µ = 0.51 mm^−^^1^
β = 96.667 (4)°	*T* = 150 K
*V* = 1844.31 (12) Å^3^	Plate, colourless
*Z* = 4	0.41 × 0.13 × 0.04 mm

### Data collection

**Table:**

SuperNova, Dual, Cu at zero, Atlas diffractometer	2866 reflections with *I* > 2σ(*I*)
ω scans	*R*_int_ = 0.043
Absorption correction: multi-scan (*CrysAlis PRO*; Agilent, 2014)	θ_max_ = 73.6°, θ_min_ = 4.6°
*T*_min_ = 0.829, *T*_max_ = 1.000	*h* = −23→23
12894 measured reflections	*k* = −12→12
3657 independent reflections	*l* = −11→11

### Refinement

**Table:**

Refinement on *F*^2^	0 restraints
Least-squares matrix: full	Hydrogen site location: inferred from neighbouring sites
*R*[*F*^2^ > 2σ(*F*^2^)] = 0.047	H-atom parameters constrained
*wR*(*F*^2^) = 0.129	*w* = 1/[σ^2^(*F*_o_^2^) + (0.0587*P*)^2^ + 0.3637*P*] where *P* = (*F*_o_^2^ + 2*F*_c_^2^)/3
*S* = 1.04	(Δ/σ)_max_ < 0.001
3657 reflections	Δρ_max_ = 0.23 e Å^−^^3^
219 parameters	Δρ_min_ = −0.17 e Å^−^^3^

### Special details

**Table:**

Experimental. Absorption correction: CrysAlisPro, Agilent Technologies, Version 1.171.36.28 (release 01-02-2013 CrysAlis171 .NET) (compiled Feb 1 2013,16:14:44) Empirical absorption correction using spherical harmonics, implemented in SCALE3 ABSPACK scaling algorithm.
Geometry. All e.s.d.'s (except the e.s.d. in the dihedral angle between two l.s. planes) are estimated using the full covariance matrix. The cell e.s.d.'s are taken into account individually in the estimation of e.s.d.'s in distances, angles and torsion angles; correlations between e.s.d.'s in cell parameters are only used when they are defined by crystal symmetry. An approximate (isotropic) treatment of cell e.s.d.'s is used for estimating e.s.d.'s involving l.s. planes.

### Fractional atomic coordinates and isotropic or equivalent isotropic displacement parameters (Å^2^)

**Table:**

	*x*	*y*	*z*	*U*_iso_*/*U*_eq_	
C1	0.28102 (8)	0.40404 (13)	0.14783 (14)	0.0259 (3)	
C2	0.18929 (7)	0.30261 (13)	0.27980 (14)	0.0246 (3)	
C3	0.26279 (8)	0.45814 (13)	0.39845 (15)	0.0272 (3)	
C4	0.29777 (8)	0.48405 (13)	0.28214 (15)	0.0273 (3)	
C5	0.28292 (9)	0.52724 (14)	0.52321 (16)	0.0314 (3)	
H5	0.2592	0.5103	0.6025	0.038*	
C6	0.33672 (9)	0.61972 (15)	0.53300 (17)	0.0356 (4)	
H6	0.3500	0.6650	0.6187	0.043*	
C7	0.37112 (9)	0.64606 (15)	0.41752 (18)	0.0358 (4)	
H7	0.4078	0.7099	0.4232	0.043*	
C8	0.35142 (8)	0.57829 (15)	0.29334 (17)	0.0326 (3)	
H8	0.3750	0.5966	0.2142	0.039*	
C9	0.12820 (8)	0.20985 (13)	0.27592 (14)	0.0259 (3)	
C10	0.11781 (8)	0.10256 (14)	0.18380 (16)	0.0293 (3)	
H10	0.1501	0.0869	0.1183	0.035*	
C11	0.06087 (9)	0.01805 (15)	0.18637 (17)	0.0347 (4)	
H11	0.0544	−0.0546	0.1225	0.042*	
C12	0.01348 (9)	0.03893 (17)	0.28127 (18)	0.0369 (4)	
H12	−0.0251	−0.0198	0.2837	0.044*	
C13	0.02269 (9)	0.14620 (18)	0.37290 (18)	0.0399 (4)	
H13	−0.0099	0.1616	0.4378	0.048*	
C14	0.07937 (9)	0.23099 (16)	0.36999 (17)	0.0346 (4)	
H14	0.0851	0.3045	0.4328	0.041*	
C15	0.26253 (8)	0.49478 (14)	0.01923 (15)	0.0286 (3)	
H15A	0.3044	0.5479	0.0049	0.034*	
H15B	0.2520	0.4366	−0.0637	0.034*	
C16	0.20181 (9)	0.59109 (15)	0.02432 (16)	0.0322 (3)	
H16A	0.2107	0.6483	0.1084	0.039*	
H16B	0.1586	0.5394	0.0319	0.039*	
C17	0.19140 (8)	0.67945 (14)	−0.10553 (16)	0.0309 (3)	
H17A	0.2352	0.7288	−0.1141	0.037*	
H17B	0.1817	0.6217	−0.1891	0.037*	
C18	0.13220 (9)	0.77946 (17)	−0.10306 (19)	0.0410 (4)	
H18A	0.0880	0.7313	−0.1029	0.061*	
H18B	0.1300	0.8369	−0.1859	0.061*	
H18C	0.1404	0.8347	−0.0187	0.061*	
C19	0.34439 (8)	0.31704 (14)	0.12029 (15)	0.0284 (3)	
H19A	0.3305	0.2605	0.0373	0.034*	
H19B	0.3820	0.3773	0.0966	0.034*	
C20	0.37407 (8)	0.22668 (15)	0.23908 (16)	0.0322 (3)	
H20A	0.3375	0.1632	0.2613	0.039*	
H20B	0.3877	0.2818	0.3233	0.039*	
C21	0.43742 (9)	0.14803 (16)	0.20321 (18)	0.0380 (4)	
H21A	0.4249	0.0997	0.1141	0.046*	
H21B	0.4755	0.2115	0.1894	0.046*	
C22	0.46367 (11)	0.0478 (2)	0.3163 (2)	0.0534 (5)	
H22A	0.4806	0.0958	0.4023	0.080*	
H22B	0.5018	−0.0049	0.2851	0.080*	
H22C	0.4255	−0.0121	0.3343	0.080*	
N1	0.22079 (6)	0.31594 (12)	0.16295 (12)	0.0269 (3)	
H1	0.2042	0.2684	0.0897	0.032*	
N2	0.20764 (7)	0.36574 (12)	0.39726 (12)	0.0281 (3)	

### Atomic displacement parameters (Å^2^)

**Table:**

	*U*^11^	*U*^22^	*U*^33^	*U*^12^	*U*^13^	*U*^23^
C1	0.0316 (8)	0.0222 (6)	0.0253 (7)	−0.0012 (5)	0.0088 (6)	0.0013 (5)
C2	0.0291 (7)	0.0207 (6)	0.0248 (7)	0.0044 (5)	0.0060 (5)	0.0031 (5)
C3	0.0328 (8)	0.0205 (6)	0.0285 (7)	0.0035 (5)	0.0049 (6)	0.0009 (5)
C4	0.0322 (7)	0.0201 (6)	0.0297 (7)	0.0024 (5)	0.0045 (6)	0.0022 (5)
C5	0.0408 (9)	0.0265 (7)	0.0273 (7)	0.0023 (6)	0.0054 (6)	−0.0007 (6)
C6	0.0445 (9)	0.0260 (7)	0.0349 (8)	0.0018 (6)	−0.0013 (7)	−0.0062 (6)
C7	0.0369 (9)	0.0247 (7)	0.0456 (9)	−0.0037 (6)	0.0032 (7)	−0.0020 (6)
C8	0.0376 (8)	0.0256 (7)	0.0357 (8)	−0.0009 (6)	0.0089 (7)	0.0010 (6)
C9	0.0297 (7)	0.0240 (6)	0.0243 (7)	0.0022 (5)	0.0049 (6)	0.0050 (5)
C10	0.0344 (8)	0.0264 (7)	0.0287 (7)	0.0016 (6)	0.0104 (6)	0.0019 (5)
C11	0.0389 (9)	0.0254 (7)	0.0404 (9)	−0.0018 (6)	0.0070 (7)	−0.0001 (6)
C12	0.0320 (8)	0.0353 (8)	0.0440 (9)	−0.0029 (6)	0.0068 (7)	0.0066 (7)
C13	0.0338 (9)	0.0490 (10)	0.0398 (9)	−0.0008 (7)	0.0168 (7)	−0.0003 (7)
C14	0.0366 (9)	0.0372 (8)	0.0313 (8)	0.0006 (6)	0.0101 (6)	−0.0039 (6)
C15	0.0349 (8)	0.0257 (7)	0.0265 (7)	−0.0016 (6)	0.0092 (6)	0.0023 (5)
C16	0.0378 (8)	0.0286 (7)	0.0313 (8)	0.0019 (6)	0.0083 (6)	0.0035 (6)
C17	0.0358 (8)	0.0264 (7)	0.0305 (8)	−0.0011 (6)	0.0036 (6)	0.0011 (6)
C18	0.0396 (9)	0.0356 (8)	0.0477 (10)	0.0043 (7)	0.0053 (7)	0.0087 (7)
C19	0.0334 (8)	0.0265 (7)	0.0267 (7)	−0.0005 (6)	0.0094 (6)	0.0009 (5)
C20	0.0380 (8)	0.0292 (7)	0.0301 (8)	0.0036 (6)	0.0075 (6)	0.0020 (6)
C21	0.0387 (9)	0.0322 (8)	0.0443 (9)	0.0047 (7)	0.0095 (7)	0.0024 (7)
C22	0.0478 (11)	0.0497 (11)	0.0628 (12)	0.0166 (9)	0.0060 (9)	0.0133 (9)
N1	0.0345 (7)	0.0247 (6)	0.0227 (6)	−0.0036 (5)	0.0084 (5)	−0.0009 (4)
N2	0.0352 (7)	0.0253 (6)	0.0248 (6)	−0.0012 (5)	0.0079 (5)	−0.0002 (5)

### Geometric parameters (Å, º)

**Table:**

C1—N1	1.4783 (18)	C13—H13	0.9500
C1—C4	1.523 (2)	C14—H14	0.9500
C1—C15	1.5432 (19)	C15—C16	1.521 (2)
C1—C19	1.5480 (19)	C15—H15A	0.9900
C2—N2	1.3079 (19)	C15—H15B	0.9900
C2—N1	1.3466 (18)	C16—C17	1.525 (2)
C2—C9	1.496 (2)	C16—H16A	0.9900
C3—C4	1.398 (2)	C16—H16B	0.9900
C3—C5	1.401 (2)	C17—C18	1.520 (2)
C3—N2	1.4078 (19)	C17—H17A	0.9900
C4—C8	1.394 (2)	C17—H17B	0.9900
C5—C6	1.385 (2)	C18—H18A	0.9800
C5—H5	0.9500	C18—H18B	0.9800
C6—C7	1.385 (2)	C18—H18C	0.9800
C6—H6	0.9500	C19—C20	1.517 (2)
C7—C8	1.389 (2)	C19—H19A	0.9900
C7—H7	0.9500	C19—H19B	0.9900
C8—H8	0.9500	C20—C21	1.526 (2)
C9—C10	1.391 (2)	C20—H20A	0.9900
C9—C14	1.397 (2)	C20—H20B	0.9900
C10—C11	1.388 (2)	C21—C22	1.523 (2)
C10—H10	0.9500	C21—H21A	0.9900
C11—C12	1.382 (2)	C21—H21B	0.9900
C11—H11	0.9500	C22—H22A	0.9800
C12—C13	1.387 (2)	C22—H22B	0.9800
C12—H12	0.9500	C22—H22C	0.9800
C13—C14	1.386 (2)	N1—H1	0.8800
			
N1—C1—C4	108.69 (11)	C1—C15—H15B	108.1
N1—C1—C15	108.52 (12)	H15A—C15—H15B	107.3
C4—C1—C15	112.35 (11)	C15—C16—C17	111.60 (12)
N1—C1—C19	109.17 (11)	C15—C16—H16A	109.3
C4—C1—C19	110.25 (12)	C17—C16—H16A	109.3
C15—C1—C19	107.80 (11)	C15—C16—H16B	109.3
N2—C2—N1	124.93 (13)	C17—C16—H16B	109.3
N2—C2—C9	116.96 (12)	H16A—C16—H16B	108.0
N1—C2—C9	118.10 (12)	C18—C17—C16	113.28 (13)
C4—C3—C5	119.01 (14)	C18—C17—H17A	108.9
C4—C3—N2	123.35 (13)	C16—C17—H17A	108.9
C5—C3—N2	117.64 (13)	C18—C17—H17B	108.9
C8—C4—C3	119.10 (14)	C16—C17—H17B	108.9
C8—C4—C1	120.17 (13)	H17A—C17—H17B	107.7
C3—C4—C1	120.61 (13)	C17—C18—H18A	109.5
C6—C5—C3	121.19 (14)	C17—C18—H18B	109.5
C6—C5—H5	119.4	H18A—C18—H18B	109.5
C3—C5—H5	119.4	C17—C18—H18C	109.5
C5—C6—C7	119.83 (15)	H18A—C18—H18C	109.5
C5—C6—H6	120.1	H18B—C18—H18C	109.5
C7—C6—H6	120.1	C20—C19—C1	116.19 (12)
C6—C7—C8	119.38 (15)	C20—C19—H19A	108.2
C6—C7—H7	120.3	C1—C19—H19A	108.2
C8—C7—H7	120.3	C20—C19—H19B	108.2
C7—C8—C4	121.49 (14)	C1—C19—H19B	108.2
C7—C8—H8	119.3	H19A—C19—H19B	107.4
C4—C8—H8	119.3	C19—C20—C21	112.19 (12)
C10—C9—C14	118.18 (14)	C19—C20—H20A	109.2
C10—C9—C2	123.21 (12)	C21—C20—H20A	109.2
C14—C9—C2	118.61 (13)	C19—C20—H20B	109.2
C11—C10—C9	120.86 (13)	C21—C20—H20B	109.2
C11—C10—H10	119.6	H20A—C20—H20B	107.9
C9—C10—H10	119.6	C22—C21—C20	112.66 (14)
C12—C11—C10	120.39 (15)	C22—C21—H21A	109.1
C12—C11—H11	119.8	C20—C21—H21A	109.1
C10—C11—H11	119.8	C22—C21—H21B	109.1
C11—C12—C13	119.48 (15)	C20—C21—H21B	109.1
C11—C12—H12	120.3	H21A—C21—H21B	107.8
C13—C12—H12	120.3	C21—C22—H22A	109.5
C14—C13—C12	120.16 (14)	C21—C22—H22B	109.5
C14—C13—H13	119.9	H22A—C22—H22B	109.5
C12—C13—H13	119.9	C21—C22—H22C	109.5
C13—C14—C9	120.92 (15)	H22A—C22—H22C	109.5
C13—C14—H14	119.5	H22B—C22—H22C	109.5
C9—C14—H14	119.5	C2—N1—C1	125.27 (12)
C16—C15—C1	116.92 (12)	C2—N1—H1	117.4
C16—C15—H15A	108.1	C1—N1—H1	117.4
C1—C15—H15A	108.1	C2—N2—C3	116.84 (12)
C16—C15—H15B	108.1		

### Hydrogen-bond geometry (Å, º)

**Table:**

*D*—H···*A*	*D*—H	H···*A*	*D*···*A*	*D*—H···*A*
N1—H1···N2^i^	0.88	2.29	3.1239 (16)	157

Symmetry code: (i) *x*, −*y*+1/2, *z*−1/2.
