# Supplementary figures and images for: Crystal structure of 4,4-dibutyl-2-phenyl-3,4-di­hydro­quinazoline
Source: Acta Crystallogr Sect E Struct Rep Online. 2014 Sep 10;70(Pt 10):o1100. doi: 10.1107/S1600536814020017 (PMC4257218; doi:10.1107/S1600536814020017)

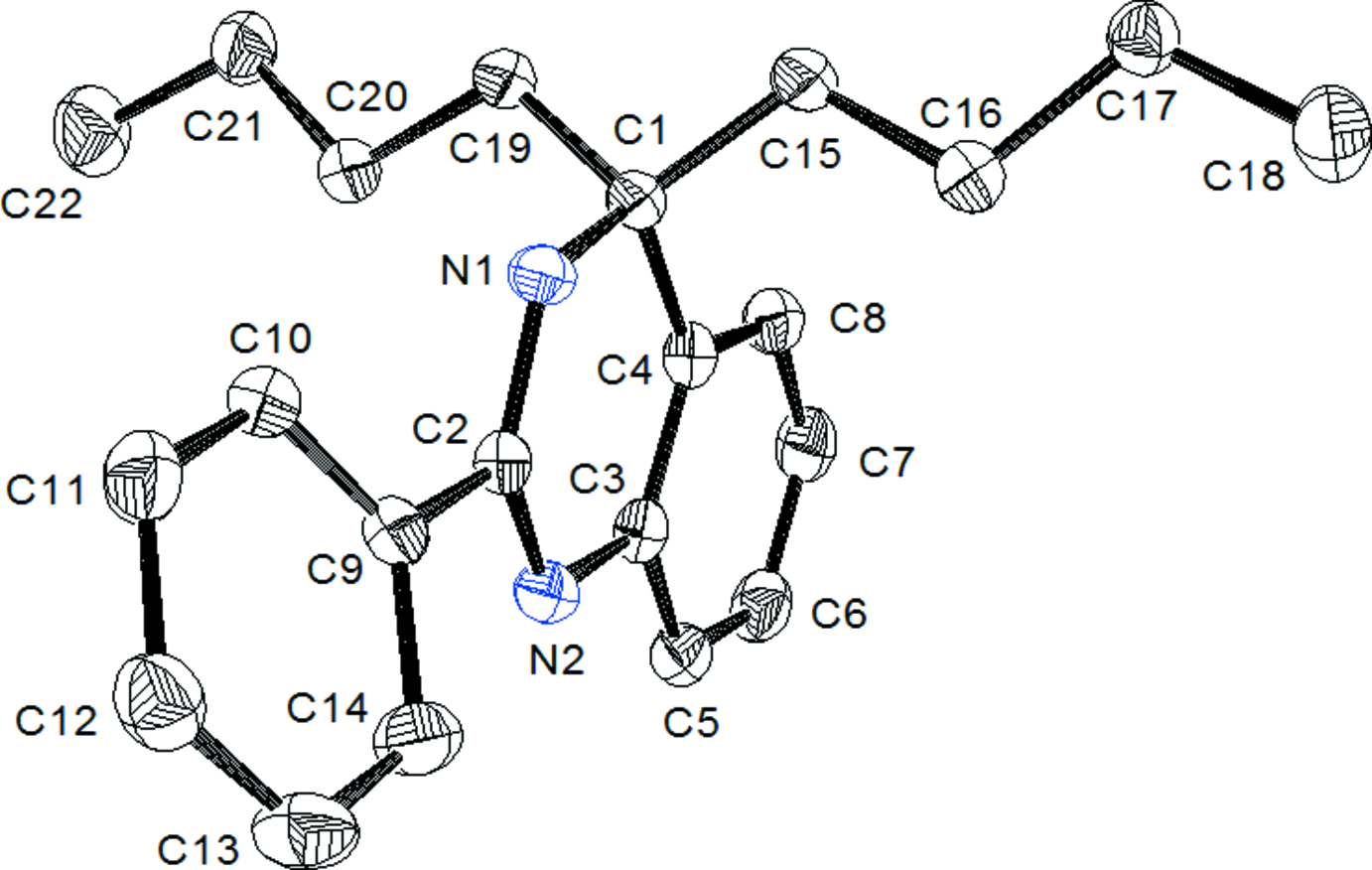

Supplement: Supplementary file 4 [file e-70-o1100-fig1.tif]

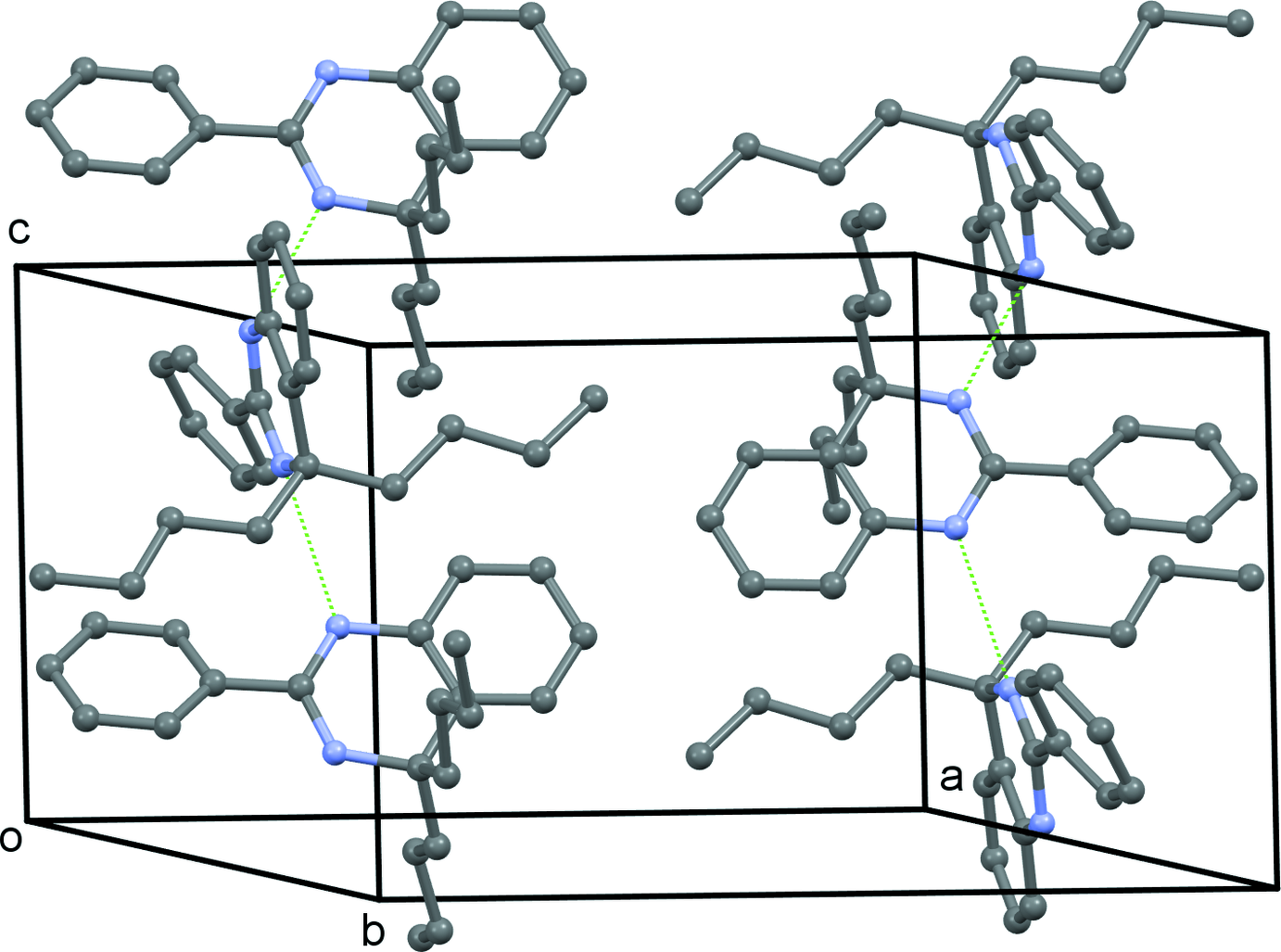

Supplement: Supplementary file 5 [file e-70-o1100-fig2.tif]
